# The Validity of a Mixed Reality-Based Automated Functional Mobility Assessment

**DOI:** 10.3390/s19092183

**Published:** 2019-05-11

**Authors:** Ruopeng Sun, Roberto G. Aldunate, Jacob J. Sosnoff

**Affiliations:** 1 Department of Kinesiology and Community Health, University of Illinois at Urbana-Champaign, Urbana, IL 61801, USA; aldunate@illinois.edu (R.G.A.); jsosnoff@illinois.edu (J.J.S.); 2 Department of Orthopaedic Surgery, Stanford University, Stanford, CA 94305, USA

**Keywords:** mixed reality headset, mobility assessment, wearable sensor, fall risk, aging

## Abstract

Functional mobility assessments (i.e., Timed Up and Go) are commonly used clinical tools for mobility and fall risk screening in older adults. In this work, we proposed a new Mixed Reality (MR)-based assessment that utilized a Microsoft HoloLens^TM^ headset to automatically lead and track the performance of functional mobility tests, and subsequently evaluated its validity in comparison with reference inertial sensors. Twenty-two healthy adults (10 older and 12 young adults) participated in this study. An automated functional mobility assessment app was developed, based on the HoloLens platform. The mobility performance was recorded with the headset built-in sensor and reference inertial sensor (Opal, APDM) taped on the headset and lower back. The results indicate that the vertical kinematic measurements by HoloLens were in good agreement with the reference sensor (Normalized RMSE ~ 10%, except for cases where the inertial sensor drift correction was not viable). Additionally, the HoloLens-based test completion time was in perfect agreement with the clinical standard stopwatch measure. Overall, our preliminary investigation indicates that it is possible to use an MR headset to automatically guide users (without severe mobility deficit) to complete common mobility tests, and this approach has the potential to provide an objective and efficient sensor-based mobility assessment that does not require any direct research/clinical oversight.

## 1. Introduction

Falls are the leading cause of injury-related death in older adults [1]. Over 1 in 4 older adults will experience a fall in the next year, and a significant portion of those that fall will suffer an injury [2], resulting in more than $50 billion in annual medical costs [3]. Moreover, although most falls do not end in death or result in a significant physical injury, they can have a psychological impact, resulting in anxiety and depression that further decreases the quality of life [4]. The risk of falling increases with aging due to multiple risk factors, such as deficits in vision, cognition, muscle strength and mobility [1]. Given the frequency and severe consequences of falls, there is a critical need for early and regular monitoring of individuals’ fall risks in order to reduce falls and fall-related injuries.

Indeed, the American Geriatrics Society (AGS) and the Centers for Disease Control and Prevention (CDC) recommends an annual fall risk screening for older adults [5,6]. The most commonly used fall risk screening tests include functional mobility tests, such as the five time sit to stand test (STS) [7] and timed up and go test (TUG) [8]. Both tests are valid and reliable clinical tests focusing on assessing lower-limb muscle strength and mobility. The STS has participants stand up and sit down from a chair five times as quickly as possible without using the armrest. Meanwhile, the TUG test requires the participant to stand up from a chair, walk 3 m at normal pace, turn around and return to the chair. 

Even though such standardized tests are relatively easy to conduct, they are still underutilized and not routinely integrated into clinical practice [9]. This is partially due to clinicians’ time constraints and competing medical priorities, a lack of accessible lab-grade advanced testing equipment (such as motion capture devices and force platforms), as well as a lack of clinical expertise [9]. Consequently, older adults remain unaware of their individual fall risk, appropriate fall prevention approaches and are at an elevated risk of falling.

With the recent advancement of sensing technology, sensor-based fall risk assessments have received a growing interest for their potential portability, accessibility and cost effectiveness [9,10]. Specifically, wearable sensors (inertial measurement unit, IMU) consisting of miniature accelerometers, gyroscopes and/or magnetometers have been used to quantify movement patterns/abnormalities [9,11]. 

Two recent systematic reviews on the sensor-based fall risk assessment [9,12] identified over 50 investigations using IMUs for the fall risk assessment in older adults. This body of literature highlights that sensors are capable of accurately quantifying mobility in high fall risk individuals. Overall, it is concluded that wearable sensors are a viable technology for fall risk assessment. It is worth noting that in most investigations, the IMU sensor(s) was attached to the lower back and/or lower limb as a stand-alone recording device, and still required additional personnel to guide the wearer through the assessment protocols. 

An alternative approach to further improve the efficiency of a sensor-based assessment is to offer a direct technology interaction with the intended users, such as delivering demonstrations and instructions, and receiving user inputs. Such a system would be able to provide an automated and self-guided assessment system that requires no additional personnel. To achieve this goal, an ideal device should be able to communicate with the wearer both through visual and auditory prompts, and allow user input through natural interactions (gesture, voice, gaze, etc.). Indeed, recent research has highlighted that smartphones and tablets can provide valid and reliable fall risk assessments to older adults [13,14]. 

**Figure 1:**
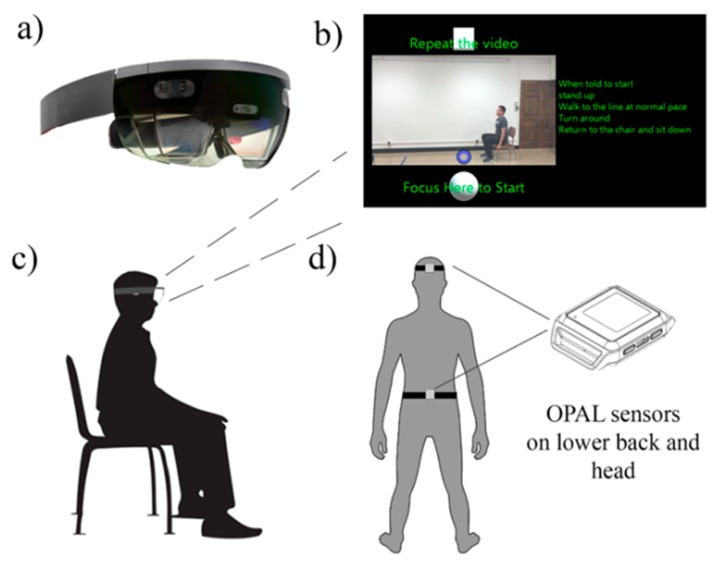
Illustration of the system setup. (**a**) HoloLens headset; (**b**) Onscreen hologram instruction for the timed up and go test (Shaded background, video animation, green font, white control button and purple gaze cursor); (**c**) Illustration of the participant’s starting position; and (**d**) Reference IMU sensors placement.

Mixed reality head-mounted display (HMD, e.g., Microsoft HoloLens, Figure 1a) systems are also uniquely fitted for technology-based mobility/fall-risk assessments. For instance, the HoloLens uses a transparent display with a light projector to provide holograms on the lenses in front of a user’s eye that blend the digital display with the physical environments [15]. It contains multiple sensors to scan the user’s environment, which enables the holograms to be placed at a specific location in the real world [15]. By using such a device in mobility assessments, the user can receive instructions and visual demonstrations, naturally interact with the virtual display through voice command, gesture control and gaze, and complete mobility tests with full visibility of the surrounding environments. In addition, the embedded IMU and depth sensor can be used to track the user’s head movement during the balance and mobility tests. Although less commonly used than sensors mounted on the lower limb and lower back for mobility assessments, head movement has been used as an approach for mobility evaluations, based on the notion that head movement is linked to the trunk movement as well as to gait-related oscillations during locomotion [16,17]. Additionally, given that head stabilization has been shown as a critical component in maintaining upright posture [16], monitoring head movement may provide novel insights into mobility control and fall risk evaluation. These unique features of HMD have the potential to enable older adults to complete fall risk screening intuitively and autonomously. 

Although this Mixed Reality Headset holds promise for enhancing the fall risk assessment in community dwelling older adults, its validity for an objective mobility assessment has not been investigated. Therefore, the aim of this study is to evaluate the validity of the mixed-reality headset for automated mobility assessments in young and older adults (denoted as YA and OA, respectively). Specifically, we aimed to evaluate the agreement of the kinematic measurement between the HoloLens, industry standard IMU sensors, and clinical standard stopwatch. We also aimed to compare the functional mobility performance difference between young and older adults.

## 2. Materials and Methods

### 2.1. Participants

Given the exploratory nature of this investigation, and based on the sample size from previous publications on novel device validation for functional assessment [18,19], twenty-two healthy adults (10 older adults (OA) and 12 young adults (YA)) participated in this study. The inclusion criteria for participation were an age between 18–30 years and 65 years or older, the ability to stand 30 s unaided, the ability to walk with or without aid, having normal or corrected to normal hearing and vision, not having a history of neuro-muscular or cardiovascular disease, and not having a history of motion sickness, chronic neck pain or seizure-related conditions. All procedures were approved by the University of Illinois at Urbana-Champaign Institutional Review Board, and all participants completed a written informed consent prior to participation. Participant testing was performed at 2 sites selected for the convenience for the participants. OAs were tested in an unoccupied apartment setting at a local retirement community, while YAs were tested in a university laboratory setting. 

### 2.2. System Setup

A custom-built Universal Windows Platform application was developed with Unity (2018 2.6 personal) and Visual Studio (Microsoft Visual Studio 2017), and deployed on the Microsoft HoloLens head-mounted display operating the Windows 10 system. The HoloLens features depth cameras for environment scanning and spatial mapping, as well as IMU for position and orientation estimation [15]. The transparent visor and light projector allow the user to see high-definition virtual content (hologram) over real world objects (Figure 1b). The field of view from HoloLens was estimated to be 30° H and 17.5 V [15]. The system can operate as a stand-alone device that requires neither a PC nor smartphone. For this project, the onscreen display was also streamed on a laptop for monitoring the participant’s interaction with the system. The HoloLens features multimodal user interaction methods, such as finger pinch, voice command, estimated gaze fixation, etc. In order to simplify the user interaction and allow an intuitive control for a senior user, we chose to use a gaze fixation (orientation estimated) to control the interface, i.e., the user will control the system by fixating their gaze on a control button for 1–2 s (Figure 1b, purple circle as gaze cursor, white blocks as control buttons. Video in Supplementary S1). Additionally, to facilitate user onboarding and ensure a self-guided test completion, the participant watched a standardized tutorial video on a laptop explaining how to put on, adjust and control the headset (Supplementary S2). Participants were encouraged to ask questions before putting on the headset. The details about the user-interface design process and usability will be reported elsewhere.

Based on the CDC fall risk assessment recommendations (Stopping Elderly Accidents, Death & Injuries—STEADI [6]) and the feasibility of head-mount movement tracking, a set of valid and reliable clinical tests, focusing on mobility and muscle strength, were selected and integrated into the automated mobility assessment app. Lower extremity muscle strength was assessed with the STS test [20,21], whereas mobility was assessed with the TUG test [8].

### 2.3. Test Procedure

Participants completed an MR-based mobility assessment, as well as a clinical fall risk assessment. During the MR-based assessment, participants were outfitted with two additional APDM Opal IMU sensors (APDM, Inc.). One was secured to the top of the HoloLens visor—denoted as the HD sensor; the other was placed on the participant’s lower back via a belt—denoted as the LB sensor (Figure 1d). After being fitted with the headset, participants were prompted to complete test sessions in the following order: (1) STS, and (2) TUG. For each test, a video recording with a standard demonstration and instruction was displayed on the headset (Figure 1b, center display and text instruction). Upon video completion, participants were provided the option to proceed to the test or to repeat the demonstration (Figure 1b, white blocks). During the test, a 5 s countdown with an audio tone and the text of the instruction was displayed. The test completion button was prompted up after a 15 s delay. After test completion, participants had options to repeat the test if not satisfied with their performance. To ensure an identical test setup between the participants, markings were placed on the ground (standing feet placement, chair location and 3 m walking path). Research personnel offered safety spotting and minimal interaction with the participant, unless asked by the participant for help. Participants who made errors during the test (i.e., a false start, incorrect number of repetitions, etc.) were asked to repeat the test.

After completion of the self-guided mobility assessment, the physiological profile assessment (PPA) [22] was administered by trained research personnel to evaluate the overall fall risk. The Montreal Cognitive Assessment (MoCA) [23] and the Activity-specific Balance Confidence (ABC) scale [24] were also administered to assess the participant’s cognitive function and balance confidence, respectively. The MoCA test is a validated screening tool for detecting cognitive impairment, whereas the ABC scale is a validated self-reported questionnaire of confidence in performing various daily activities without losing balance. The PPA consists of a set of comprehensive tests assessing vision, lower limb sensation, muscle strength, reaction time and balance, which are associated with the risk of falling [22].

### 2.4. Data Processing

Due to the HoloLens API setup on sensor data access, the raw accelerometry/gyroscope data was not accessible, and thus only the processed head position and orientation was available for recording at a dynamic sampling rate at/around 30 Hz (variation due to the windows internal clock frequency). Such data was processed through its internal proprietary sensor fusion algorithm (IMU, depth and environmental cameras), which output the 3D head spatial coordinate and gravity aligned orientation. The acceleration and gyroscope data from the Opal sensor was recorded at 128 Hz, and the gravity was corrected after the orientation estimation using an extended Kalman filter provided by APDM. Both the HoloLens and Opal data were segmented and synced to each task, resampled at 30 Hz and low-pass filtered (4th order Butterworth) with a cutoff frequency at 5 Hz [25,26,27].

For the STS and TUG tests, the gravity corrected vertical (VT) acceleration data from the Opal sensors were double integrated over time to obtain the vertical displacement, with the drift and integration error corrected using (1) a high pass filter (4th order Butterworth, 0.1 Hz) [27] and (2) a drift correction under the assumption that participants reach the same height when they make contact with the chair (zero displacement update-ZDU) [18]. The processed VT displacement from the HoloLens and Opal sensors was then time-aligned using a cross correlation analysis (calculating the similarity and time lag between the signal). Finally, time-aligned VT kinematic data (displacement, velocity, and acceleration) profiles were derived from Opal and HoloLens using numerical integration and differentiation accordingly. The VT data were also used to calculate the following performance features: (1) the STS duration: the time between the initiation of the first chair rising and the completion of the last chair descending [25,26]. (2) the STS mean duration of the sitting and standing phase [25,26]. (3) The maximum acceleration and velocity during STS. (4) The TUG duration: time between the initiation of the chair rising and the completion of the chair descending. (5) The maximum acceleration and velocity during TUG. Due to a significant signal drift over time in the AP, ML direction using the Opal sensor (see discussion), and a lack of a viable signal correction method, the signal comparison in the AP/ML direction was not performed for the STS and TUG tests. 

The pairwise signal agreements between HoloLens, HD and LB sensors were analyzed using the normalized root mean squared error (NRMSE – RMSE divided by the signal amplitude range), as well as the cross-correlation coefficient (correlation coefficient at zero lag, denoted as Xcor). The NRMSE reports the error as a ratio of the measurement range, with lower values indicating a better signal agreement [19]. The Xcor measures the signal similarity of two time series, with a higher value (~1) indicating a better signal agreement [27]. The mean and 95% confidence interval of NRMSE and Xcor were calculated through the functional test condition (STS, TUG). 

The STS and TUG duration, derived from the HoloLens measure, was compared with the manual stopwatch tracking using a Bland-Altman limit of agreement analysis [28]. The Bland-Altman limit of agreement is a robust statistical approach to indicate the level of agreement between any two measurements. Since a high correlation between any two methods does not necessarily mean that the two methods are in good agreement, the Bland–Altman technique is utilized in many studies to investigate the presence of an absolute agreement between the two technologies. 

For all derived mobility features from the HoloLens (STS duration, STS mean duration of the sitting and standing phase, maximum acceleration and velocity during STS, TUG duration, and maximum acceleration and velocity during TUG), a group (OA and YA) comparison was also conducted using a two-tail student t-test. All data processing and statistical analyses were performed with a customized MATLAB program (MathWorks, Inc., Natick, MA, USA)

## 3. Results

Two older participants (82 and 91 years old, both male and having a high fall risk—PPA >2) did not complete the self-guided assessment, due to balance/mobility deficits and the need for assistance in challenging conditions. Therefore, only 8 OA and 12 YA were included in the data analysis. It is worth noting that only 1 of the remaining 8 OA had a high fall risk (PPA > 2). Additionally, 2 OA participants were asked to repeat the test due to a performance error (false start, wrong number of sit to stand repetitions, etc.). The participants’ demographic characteristics and physiological profile, measured by PPA, are presented in Table 1. As expected, a significant difference in age, cognitive function (MoCA), contrast visual acuity (MET), reaction time, muscle strength, and overall risk of falls was observed between OA and YA. 

**Figure 2:**
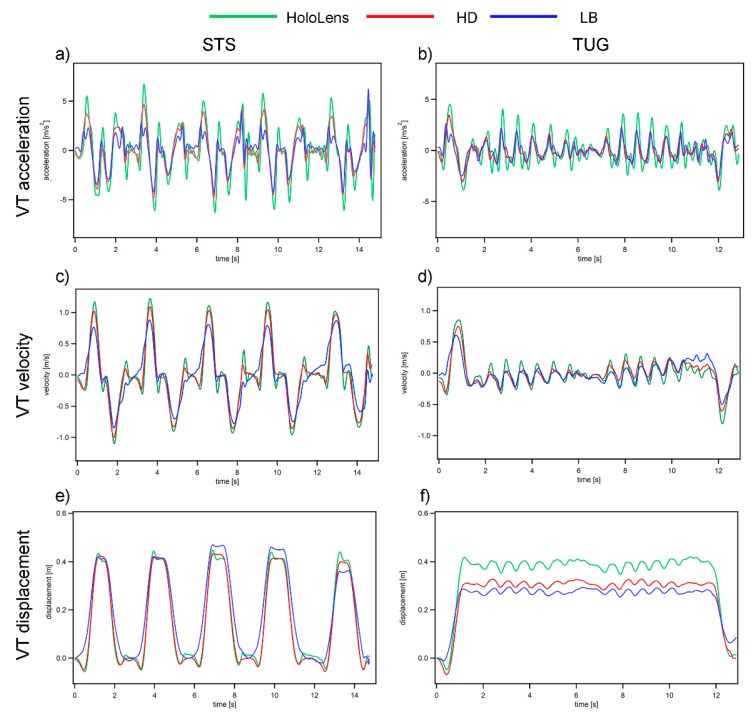
Sample kinematic profile from a young participant. Green denotes HoloLens, red denotes HD sensor, and blue denotes LB sensor. (**a**,**b**) VT acceleration profile from the STS and TUG tasks; (**c**,**d**) VT velocity profile from the STS and TUG tasks; and (**e**,**f**) VT displacement profile from the STS and TUG tasks.

Representative data traces of the STS and TUG test from a young participant (27 years old, female) are illustrated in Figure 2. A vertical kinematic data comparison between the HoloLens and Opal sensors using NRMSE and Xcor are presented in Table 2. Overall, the data recorded from HoloLens (green line in Figure 2) is in good agreement with the HD/LB sensors (red/blue line in Figure 2). In general, the signal similarity as measured by the Xcor is good to excellent (0.740–0.998), with a higher Xcor observed in the displacement measure (0.965–0.998) in comparison to the velocity (0.853–0.979) and acceleration (0.740–0.888). This finding can be explained as the displacement drift was corrected using the ZDU method, whereas the velocity and acceleration signal from the HD/LB sensors remained slightly affected by the integration/differential error. For the STS task, the signal agreements as measured by NRMSE were excellent (below 10%) for all of the measures except for the displacement comparison between the HoloLens and LB sensors (11.88%). On the other hand, for the TUG task, the signal agreement was relatively low (close to 20% NRMSE for the displacement comparison), which is likely due to the fact that the ZDU drift correction was only performed once over the entire recording for the HD/LB sensor (contrary to the drift correction after each sitting cycle in the STS task), resulting in a higher vertical displacement bias (Figure 2e).

**Figure 3:**
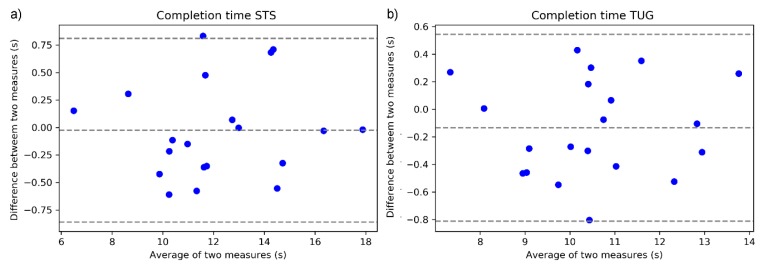
Bland-Altman plot of the sensor-derived and stopwatch timed task completion time. (**a**) STS completion time; and (**b**) TUG completion time. The *Y*-axis indicates the difference between the measures (a positive value indicates that the stopwatch measure is larger than the sensor-derived measure).

Figure 3a,b shows the Bland-Altman plot for the agreement in the STS and TUG completion time between HoloLens and the manual stopwatch recording. The absolute difference between each data pair is plotted against their mean. The two horizontal lines represent the 95% limits of agreement (range of error) calculated as 1.96 times the standard deviation from the mean differences between the two methods. The figure illustrates that the mean difference between the two methods is less than 0.02 s for STS and 0.13 s for the TUG measure, with range of error within ±0.8 s.

Table 3 shows the group comparison (OA vs. YA) in functional performance outcomes derived from the HoloLens measures. Overall, there is no significant difference between the groups, with a marginal significant difference observed in the max velocity in the STS and TUG tasks, reflecting the healthy nature of the OA sample (only 1 out of 8 participants who completed the assessment have a significant fall risk).

**Figure 4:**
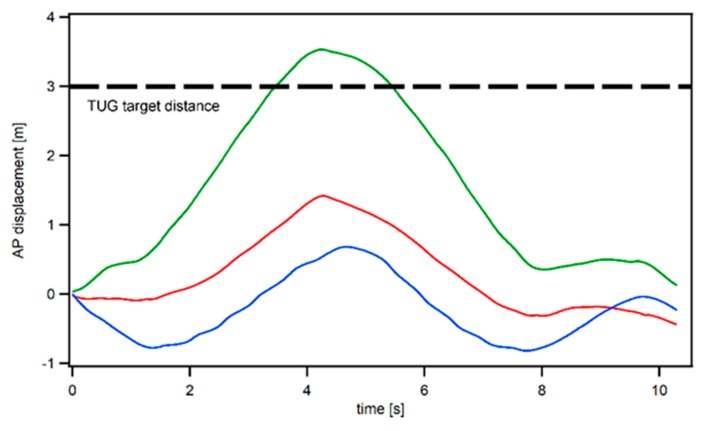
The sample AP displacement in the TUG task. Red denotes the HD sensor, and blue denotes the LB sensor. Note that only the HoloLens AP displacement measure correctly matches the 3 m walking path utilized in the TUG task.

Although the AP displacement comparison between the sensors was not analyzed statistically (due to a lack of a viable drift correction method for the IMU sensors), our exploratory investigation found that only the HoloLens AP displacement measure matches the standard 3 m walking distance utilized in the TUG, whereas the IMU derived AP displacement measure severely underestimates the walking distance (evident by the displacement measure in the AP direction, as shown in Figure 4).

## 4. Discussion

The Mixed Reality headset holds great promises for enabling a portable, self-guided mobility assessment that can be undertaken more regularly without clinician oversight, and that can subsequently increase the efficiency of current healthcare practice. This investigation is the first to evaluate the validity of MR headset (HoloLens) for mobility assessments in young and older adults. Given the unique advantage of multi-modal user interaction methods (visual/audio/gesture/gaze), this device enables users to initiate and complete a set of valid mobility assessments with step-by-step guidance, and to record head movement as a mean for an objective measure of performance.

Overall, our preliminary investigation indicates that it is possible to use a mixed reality headset to automatically guide both young and old users to complete common functional mobility tests (TUG and STS), with a good measurement accuracy in comparison to industry standard inertial sensors. More specifically, by comparing the vertical kinematic measurements (displacement, velocity, and acceleration) derived from the HoloLens and Opal sensors, we found a good to excellent signal agreement for the majority of STS and TUG measures (Xcor 0.74–0.99, NRMSE ~10%, except for the displacement in TUG), with a better signal agreement observed in the STS task, as each sit-to-stand cycle allows for a displacement calibration adjustment (Zero Displacement Update) [18]. For the sensor signal comparison in the TUG task, however, due to the integration and drift error associated with the IMU sensor and due to the lack of a viable calibration during the walking period (10 s or longer), the vertical displacement derived from the IMU sensors was biased in comparison to the HoloLens output (Figure 2e), resulting in unsatisfactory NRMSE measures (14.07–19.56%). Moreover, because of the fact that HoloLens utilized both the depth sensor and the IMU sensor to derive the displacement measure (the depth sensor on HoloLens is similar to the Kinect sensor, which has been extensively validated for accuracy in kinematic measurements [29,30]), we would expect the HoloLens output in the displacement measure (The IMU measurement corrected by the environmental scanning depth data) to be more trustworthy than the IMU derived displacement measure. Although the AP/ML displacement comparison between the sensors was not analyzed due to a lack of a viable drift correction method for the IMU sensors, our exploratory investigation (Figure 4) found that only the HoloLens AP displacement measure matches the standard 3 m walking distance utilized in the TUG, further indicating the potential superiority of HoloLens in comparison to the IMU sensors for accurate kinematic measurements.

Additionally, the sensor-derived completion time of STS and TUG was also compared with the current standard manual stopwatch method [6], using the Bland-Altman agreement plot. We found an excellent agreement with the stopwatch timing for both the STS and TUG completion times, with the HoloLens measure demonstrating less than 0.2 s in measurement bias and less than 0.8 s in range of error. It is also worth noting that the operator reaction time for the stopwatch could contribute to its measurement error. 

Because of the relatively healthy nature of the OA participant who completed the test without any assistance (only 1 OA participant has a significant fall risk, PPA >2), no group difference for the sensor-derived performance measure was found between YA and OA, with only a marginal difference observed in the maximum ascending velocity in the STS and TUG tasks. The two excluded OA participants who had a high fall risk, however, could not complete the STS and TUG tasks without requiring physical assistance, indicating the stand-alone HoloLens device and self-guided functional mobility tests may not be suitable for those with severe mobility deficits. 

We acknowledge certain limitations for this investigation, most of which are related to the pioneering use of a novel technology. First, due to the lack of optical motion tracking equipment for portable/community testing, industry standard IMUs were utilized for this validation study. Therefore, due to IMU’s inherent integration/drift error and the limited data access to the HoloLens displacement data, a horizontal (AP/ML) kinematic measurement comparison was not conducted. Second, both the relatively small sample size and the healthy nature of OA participants who can complete the functional tests without any assistance, preclude the investigation detecting the diagnostic power of using a head-mount device for fall risk/mobility deficit screening in older adults. It is possible that this novel device may only be appropriate for OA without severe fall risks who can complete functional mobility tests independently. Therefore, future studies should incorporate optical motion tracking and larger heterogeneous samples to investigate the use of HoloLens for fall risk screening. Another limitation in this work is the requirement for research personnel to place tape marking on the walk path and identify user errors, which affects the ability of this device to be used fully automatically. Future software development will integrate automatic marking of foot placement, and walking path identification using HoloLens depth sensing, as well as an onboard real-time error detection to provide a truly automatic assessment system to the user. For users with severe mobility deficits, however, an additional safety personnel should still be recommended.

The conclusion from this investigation is that the HoloLens measurement is comparable to the industry standard IMU sensor for kinematic measurements. And given its unique novelty in being able to provide video/audio instruction on the headset and record head movement during testing, we believe that it has certain potentials to be used as a stand-alone device for automated functional mobility assessments without any additional burden on research/clinical personnel. Although at the current stage, due to the device’s heaviness and fit, not all older adults can complete the required tests while wearing it (especially those who already have severe mobility deficits), this device still holds a potential for objective mobility screening among the community-dwelling aging population. It is our belief that this device could serve as a triage tool for fall risk screening in the future (focused on those who can complete the tests independently but may still have an elevated fall risk, rather than on those who cannot complete the tests and have a significant fall risk).

## Figures and Tables

**Table 1:** Participant characteristics (mean and standard deviation). * indicates the significant group difference (*p* < 0.05).

	OA n = 8,6 F	YA n = 12, 6 F
Age (yrs) *	78.2 (6.1)	24.4 (3.9)
BMI (kg/m^2^)	23.9 (3.6)	24.5 (2.9)
MoCA *	26.2 (2.3)	28.6 (1.7)
ABC	88.8 (13.3)	96.0 (3.7)
MET *	19.9 (1.5)	21.2 (0.6)
RT (ms) *	257.7 (33.6)	217.5 (32.8)
Proprio	3.0 (1.2)	3.3 (3.5)
KneeMax (kgf) *	25.3 (9.6)	41.9 (8.5)
AP sway (mm)	27.2 (9.8)	20.8 (10.9)
ML sway (mm)	33.7 (18.9)	20.5 (12.3)
PPA *	0.9 (0.7)	−0.3 (0.7)

**Table 2:** Kinematic measurement (VT acceleration, velocity, and displacement) agreement between the HoloLens and HD/LB sensors. NRMSE-Normalized Root Mean Squared Error. Xcor-Cross Correlation Coefficient. All values reported as the mean and 95% confidence interval.

**HoloLens vs. HD**				
		**A**	**V**	**D**
**STS**	**NRMSE**	9.60 (8.70,10.51)	4.83(4.22,5.45)	5.58 (4.29, 6.87)
	**Xcor**	0.888(0.872,0.904)	0.979(0.975,0.983)	0.993 (0.989 0.997)
**TUG**	**NRMSE**	10.53 (9.60,11.46)	6.16 (5.57,6.76)	19.56 (17.24,21.87)
	**Xcor**	0.802 (0.770,0.834)	0.926(0.918,0.934)	0.998 (0.997 0.999)
**HoloLens vs. LB**				
		**A**	**V**	**D**
**STS**	**NRMSE**	9.77 (8.29,11.25)	8.55 (6.98,10.12)	11.88 (9.72,14.03)
	**Xcor**	0.765 (0.704,0.827)	0.900(0.851,0.949)	0.965 (0.949,0.982)
**TUG**	**NRMSE**	8.48 (7.56,9.41)	7.68 (7.05,8.31)	14.07 (11.86,16.28)
	**Xcor**	0.740 (0.695,0.786)	0.853 (0.835,0.872)	0.986 (0.978 0.993)

**Table 3:** Group differences of key outcome measures (mean and standard deviation).

Task	Outcome Measures	OA	YA	*p*
**STS**	Total Time (s)	12.22 (3.61)	12.08 (1.99)	0.922
	Mean Stand Time (s)	0.52 (0.18)	0.64 (0.22)	0.198
	Mean Sitting Time (s)	1.15 (0.55)	1.03 (0.23)	0.575
	Max Acceleration (m/s^2^)	4.75 (1.81)	6.22 (2.03)	0.108
	Max Velocity (m/s)	1.02 (0.16)	1.20 (0.28)	0.087
**TUG**	Total Time (s)	10.61 (2.37)	10.56 (1.00)	0.96
	Max Acceleration (m/s^2^)	3.98 (0.92)	3.97 (0.62)	0.961
	Max Velocity (m/s)	0.69 (0.10)	0.81 (0.15)	0.059

## Supplementary Materials

The following are available online at https://www.mdpi.com/1424-8220/19/9/2183/s1, Video S1: Demonstration recording of the MR-based TUG task. Video S2: Tutorial video shown to the user on how to put on and adjust the headset.

## Abbreviations

IMU	Inertial Measurement Unit
HMD	Head-mounted Display
MR	Mixed Reality
MoCA	Montreal Cognitive Assessment,
ABC	Activity-specific Balance Scale
STS	Five time Sit to Stand test
TUG	Timed Up and Go test
PPA	Physiological Profile Assessment
RT	Reaction Time
MET	Melbourne Edge Test
Proprio	Proprioception
KneeMax	Maximal isometric knee extension force
AP	Anterior Posterior
ML	Medial Lateral
VT	Vertical
OA	Older Adults
YA	Young Adults
HD	Head IMU sensor
LB	Lower Back IMU sensor
A	Acceleration
V	Velocity
D	Displacement
NRMSE	Normalized Root Mean Squared Error
Xcor	Cross Correlation Coefficient
ZDU	Zero Displacement Update
